# Dynamic postural control in women athletes with and without nonspecific low back pain with high and low pain-related anxiety- A case-control study

**DOI:** 10.1186/s13102-023-00764-7

**Published:** 2023-11-07

**Authors:** Zahra Amerian, Shabnam ShahAli, Zahra Sadat Rezaeian, Sanaz Shanbehzadeh

**Affiliations:** 1 Iranian Center of Excellence in Physiotherapy, Rehabilitation Research Center, Department of Physiotherapy, School of Rehabilitation Sciences, Iran University of Medical Sciences, Tehran, Iran; 2 Musculoskeletal Research Center, Rehabilitation Research Institute and Department of Physical Therapy, Faculty of Rehabilitation Sciences,, Isfahan University of Medical Sciences, Isfahan, Iran

**Keywords:** Pain-related anxiety, Postural balance, Low back pain, Athlete

## Abstract

**Background:**

Low back pain is common among athletes and it has been shown that postural control is altered in the general population with nonspecific low back pain (NSLBP). Psychological factors may also predispose individuals to risk of altered postural control. Dynamic postural control is essential to the performance of athletes. This study aimed to compare the dynamic postural control between women athletes with and without NSLBP with high and low pain-related anxiety.

**Methods:**

Forty-five female athletes (15 NSLBP with high pain-related anxiety, 15 NSLBP with low pain-related anxiety, and 15 healthy (control)) were included. Pain-related anxiety was assessed using the Pain Anxiety Symptom Scale-20 (PASS-20). Based on the cut-off score of 30 for the total score of PASS-20, NSLBP patients were classified into two groups of low and high pain-related anxiety. Participants performed double-leg vertical drop jump (DVJ) and single-leg vertical jump (SVJ) tests on a Kistler force plate (type 9260AA6, Kistler Instruments Inc, Switzerland). The total root mean square (RMS) of the center of pressure (COP), COP displacement in the anteroposterior (AP), and mediolateral (ML) directions, COP mean velocity, and time to stabilization (TTS) in vertical, AP, and total directions were extracted from COP and ground reaction force data using MATLAB software. One-way Analysis of variance (ANOVA) and Welch’s ANOVA were employed to compare the groups. In case of significant findings, post hoc tests were performed.

**Results:**

The results showed that during DJV, athletes with high pain-related anxiety had significantly greater TTS in all total, AP, and ML directions than other groups (P < 0.05). Also, the control group showed greater total RMS distance during DJV than either NSLBP group. However, no significant differences in TTS and COP parameters were found between the groups during SVJ (P > 0.05).

**Conclusions:**

The findings suggest that pain-related anxiety may contribute to athletes’ postural control strategies. Therefore, it is important to consider the level of pain-related anxiety during planning postural control exercises for women athletes with NSLBP.

## Introduction

Low back pain (LBP) is one of the most common musculoskeletal disorders in the general population, with lifetime prevalence rates reported as high as 80% [1, 2]. This prevalence rate is almost attributable to high-performance athletes, as well. Almost 33–84% of top-level athletes experience an episode of LBP at least once during their sporting careers, despite their high physical fitness level and intensive strength training program [3, 4]. Clinical experience has shown that pain often remains after an athlete’s recovery and may also limit their participation [5].

Optimal postural control is essential for all sporting activities, including changing the body position and maintaining and implementing skills and techniques during challenging situations such as sudden changes in the direction of the ball or the path [6]. Studies have demonstrated that individuals with nonspecific low back pain (NSLBP) have poorer postural control [7–9]. In Addition, postural control impairments are observed in pain-free individuals who had an experience of LBP during the asymptomatic periods. These persistent postural changes may be due to the fear of pain [10]. It is believed that psychological mechanisms are more likely to lead to the development of NSLBP [11].

Evidence indicates the existence of neural links between the brain regions, responsible for emotion and balance [12–14]. Notably, the amygdala and limbic structures, which are critical factors in processing emotions such as anxiety, are linked via efferent projections to the brain regions involved in postural control [12]. Therefore, psychological factors such as stress, fear, and anxiety might predispose individuals at risk of altered postural control [11].

Seen in this light, the fear-anxiety avoidance model probably is the most important theory, which explains the role of psychological factors in the development of chronic disability in patients with LBP [15]. Regarding this model, patients with high levels of pain-related anxiety may anticipate pain during activities, which might lead to altered motor behavior. Hence this change might induce inappropriate spine loading, and persistent LBP [16].

Athletes could experience higher levels of emotional distress and catastrophic thinking than the general population, due to perceiving external (coaches, peers, family, and media) or internal (athletic identity, financial pressures, guilt) pressures [17]. Thus, evaluating the impact of psychological factors such as pain-related anxiety on postural control is of great importance.

Dynamic postural control assessment is more important in athletes since dynamic tests are more similar to their sporting activities such as double-leg vertical drop jump (DVJ) and single-leg vertical jump (SVJ). Dynamic postural stability has been defined as maintaining balance while transitioning from a dynamic to a static state. Postural control evaluates body sway by utilizing the force plate and measuring the sway amplitude of an individual’s center of pressure (COP) and ground reaction forces (GRF). The COP displacement provides information about the spatio-temporal aspects of postural sway and has been widely used to identify dimensions of static postural instability. In comparison with traditional stability measures, time to stabilization (TTS) is a dynamic measure of postural sway and potentially a more functional measure. TTS measures the time required to reach stability after landing, therefore it’s a measure of the transition from a dynamic task to a static state. Also, it is a general reflection of the sensory-motor system and shows the relation between LBP and central patterning [18].

Women generally land with higher peak vertical and posterior GRFs compared to men, as well as performing playing actions with lower hip flexion, hip abduction, knee flexion, and knee abduction [19, 20]. Moreover, women exhibit higher hip and knee loading in the frontal plane compared to men [21]. These sex differences in landing kinetics and kinematics increase injury potential in women athletes [19].

Previous studies have examined the relationship between spinal movement, muscle imbalance, fatigue, and postural control in athletes with and without LBP [22–26]. Likewise, several studies showed the effect of psychological factors such as fear of movement and anxiety on pain and physical function after athletes’ injuries [17, 27], but no study has been conducted to define the effect of psychological factors on postural control in the women athletes with and without NSCLPB. Therefore, the purpose of this study was to comparison of dynamic postural control between women athletes with and without NSLBP with high and low pain-related anxiety. It is hypothesized that NSLBP women athletes with high and low pain-related anxiety would demonstrate altered dynamic postural control as compared with women athletes without NSLBP.

## Method

### Study design

This case–control study was performed in the physiotherapy department of the Iran University of Medical Sciences laboratory between October 2021 and November 2022. The study was approved by the Human Research Ethics Committee of the Iran University of Medical Sciences (IR.IUMS.REC.1398.1158) and all participants signed an informed consent form.

### Participants

The sample size was calculated based on pilot data gathered from 7 athletes in each group, using the G*Power software (version 3.1.9.4) and considering the TTS during the DVJ task. From a priori analysis, a power of 0.90, α = 0.05, and an effect size f of 0.6 were set. A sample size of 39 was calculated. Considering an attrition rate of 10%, the sample size of 45 (15 in each group) was considered.

Forty-five female athletes (15 NSLBP with high pain-related anxiety, 15 NSLBP with low pain-related anxiety, and 15 healthy (control)) aged between 18 and 40 years [28] were enrolled in this study. Inclusion criteria for NSLBP were persistent pain for more than 12 weeks or at least three self-reported recurrent pain episodes during the year before testing [29] and the location of the pain was between the 12th rib and the lower gluteal fold [30]. Subjects were included when their pain was less than 30 mm on a 100-mm Visual Analogue Scale (VAS) [30] and their score on the State-Trait Anxiety Inventory questionnaire (STAI) was lower than 40 for trait anxiety. Also, participants’ state anxiety score on the STAI, as transient feelings of insecurity at the moment of testing should not be more than 40 [31].

The athletes were eligible for inclusion if they regularly participated in level I (jumping, cutting, and pivoting sports like handball or basketball) or level II sports (requiring less jumping or hard cutting than level I, like volleyball, or gymnastics), according to the criteria described by Hefti et al. [32]. All participants were self-reported collegiate-level athletes being active competitively, defined as exercising at least 6 h a week [33]. Individuals with a history of vision, vestibular, cognitive, or anxiety disorder [34], previous spinal surgery, any specific pathology of the spine, acute radicular pathology [35], recent (6 months) musculoskeletal problems in lower limbs [34], history of receiving physical-psychological treatments such as cognitive behavioral therapy due to back pain [36] were excluded. Also, none of them took medications that could alter postural control or cognitive processing [17].

NSLBP patients were classified into two groups of low and high pain-related anxiety based on the cut-off score of 30 for the total score of Pain Anxiety Symptom Scale-20 (PASS-20) [37]. Each group of NSLBP consisted of 15 participants, while the three groups were matched according to age, height, and weight.

### Measurements

#### Questionnaires

The STAI questionnaire was used at the beginning of the study to assess participant’s anxiety. This scale is a self-report psychological inventory composed of 40 questions based on a 4-point Likert scale. STAI measures two kinds of anxiety: state anxiety, or anxiety about an event, and trait anxiety, or anxiety level relative to an individual. Each subscale is scored from 20 to 80 with higher scores indicating greater anxiety level [38].

Pain-related anxiety was assessed using the PASS-20 [37]. The scale is a self-administered questionnaire consisting of 20 items that are rated on a 6-point Likert scale (0 = never, 5 = always). The total score ranges from 0 to 100, with higher scores indicating greater pain-related anxiety. The tool indicates good reliability and validity in LBP subjects [37].

Pain intensity was assessed by the Visual Analogue Scale (VAS). The scale is a self-administered measure, ranging from 0 points which indicates “no pain” to 10 points which indicates “maximum intensity of pain” [39].

All three groups filled out STAI and PASS-20 questionnaires and VAS was filled out by the NSLBP athletes with low and high pain-related anxiety.

#### Force plate

A Kistler force plate (type 9260AA6, Kistler Instruments Inc, Switzerland) was used to measure the total root mean square (RMS) of COP data, COP displacement in the anteroposterior (AP), and mediolateral (ML) directions, COP means velocity, and TTS in vertical, AP and total directions [40]. These variables represent spatiotemporal aspects of postural control and have been widely used in athletes to identify dimensions of postural instability [41–43].

### Procedure

The questionnaires and demographic data form were provided before data collection. For the preparation trial, participants warmed up for about 5 min and were familiarized with the tasks but not to the extent that it had the effect of learning [44]. All participants wore fitting athletic clothing and comfortable running shoes. Two different conditions, DVJ and SVJ were performed. Each condition consisted of 3 trials. Each trial lasted 25 s with a rest period of 60 s between trials and 5 min between conditions.

For the DVJ, participants took a bipedal hip-width stance on a 30 cm high plyometric box (positioned right next to the force platform) and were instructed to drop straight down off the box (similar to falling from a height). As soon as the sound of the beep was heard, the participant landed on both feet on the force plate, quickly jumped vertically as high as possible, and finally preserved balance for 10 s. Arm movement was not restricted during either task but at the end of the landing, hands must be placed on hips [45]. Subjects were instructed to jump to a submaximal self-selected height and, after landing, to stand as still as possible until they received a verbal signal marking the end of the trial.

For SVJ, the participant stood on both feet with hands hanging at the sides and with the toes behind a line on the ground which was located half of the participant’s height from the force plate. Participants Jumped off the double leg in the forward direction as much as possible, landed on a single dominant leg on the force plate, and maintained the position for 10 s. After landing, the hands needed to be re-positioned on the hip, while focusing a cross on the wall at the level of sight [46]. Unsuccessful trials were categorized as landing errors (touching the ground with the free leg, leaving the force plate, touching the ground with the hands and falls, and landing on the wrong foot) [44].

### Data processing

The average values of three postural tasks were used for analysis. Force plate data were sampled at 1000 samples/s. Raw data were bi-directionally filtered with a 4 Hz fourth order (2 × 2nd order) Butterworth low-pass filter at a cut-off frequency of 10 Hz. MATLAB software (MATLAB R2018, The Mathworks, Inc., Natrick, RI, USA) was used for data processing. The Primary outcomes were TTS in vertical, AP, and total directions [40]. To calculate the TTS, the GRF in the vertical, AP force, and the overall force vector (including all three forces in three dimensions) were used. The total period that the test was taken was divided into 500-millisecond intervals, which is marked on the graph as a window. The peak and minimum levels of the GRF were obtained for each interval, and the force change was calculated. An unbounded third-order polynomial is selected and set as the stable range. An unbounded third-order polynomial was fit to the rectified data from the peak GRF, for each direction of GRF. A horizontal line is drawn from the point of peak GRF value of the selected interval to the polynomial. The point in time when the polynomial intersected with the line was considered the TTS [47]. The secondary outcomes were COP parameters (COP peak-to-peak range of displacement in ML and AP directions, total RMS distance, and mean velocity) calculated during the 10-second interval after landing.

### Statistical analysis

The SPSS software (Version 26) was used for data analysis. The normality of the data was verified using the Shapiro– Wilk test and the homogeneity of variance by the Levene test. As all of the variables followed a normal distribution, a parametric one-way analysis of variance (ANOVA) was performed. Since unequal variance was detected by Levene’s Test for TTS parameters during DVJ, Welch’s ANOVA was carried out to compare the three groups. In case of significant findings, post hoc analysis was carried out; Bonferroni’s multiple comparisons for equal variances; and the Games-Howell post hoc test for unequal variances. The αlevel was set at 0.05 for each analysis.

## Results

Demographic characteristics are presented in Table 1. There were no significant differences between the groups related to age, height, weight, and Body Mass Index (BMI) of the subjects. The NSLBP group with high pain-related anxiety had significantly higher PASS-20 and STAI scores than the other two groups (NSLBP with low pain-related anxiety and control groups) (*p* < 0.05). The PASS-20 and STAI scores were not different between NSLBP with low pain-related anxiety and control groups. There were no significant differences in VAS scores between NSLBP athletes with high and low pain-related anxiety (*p* ˃ 0.05).

**Table 1:** Demographic and descriptive information

Variable	Group			*p*-value
	Control	NSLBP with low pain-related anxiety	NSLBP with high pain-related anxiety	
**Age (year)**	23.80 (5.46)	22.86 (2.89)	22.93 (3.53)	0.78
**Height (cm)**	163.06 (7.17)	170.26 (8.96)	168.53 (9.19)	0.06
**Weight (kg)**	53.36 (9.19)	62.36 (11.09)	59.66 (10.38)	0.06
**PASS-20**	15.33 (4.53)	17.06 (4.93)	35.53 (2.71)	0.00*
**STAI (Trait anxiety)**	23.86 (3.24)	28.06 (6.52)	36.60 (4.04)	0.00*
**STAI (STATE anxiety)**	23.86 (3.84)	28.06 (6.52)	36.60 (4.04)	0.00*
**VAS**		1(0.84)	1.26 (0.59)	0.00*

NSLBP: nonspecific low back pain, PASS-20: pain anxiety symptom scale, STAI: State-Trait Anxiety Inventory questionnaire, VAS: Visual Analogue Scale

### Dynamic stability parameters

#### TTS parameters

Table 2 summarizes the results of the ANOVA. There was no significant difference between groups in all TTS parameters during SVJ.

**Table 2:** Pair-wise comparison of the dynamic stability and postural hsway parameters between groups

Outcome	Group	SVJ mean (SD)	DVJ mean (SD)	Pairwise comparison during SVJ	*p*-value	Pairwise comparison during DVJ	*p*-value
**Dynamic stability**							
TTS vertical (s)	NSLBP-High A	2.08(0.18)	2.94(0.57)	NSLBP-High A /Control	1.00	NSLBP-High A /Control	0.00
	NSLBP-Low A	2.09(0.12)	2.47(0.17)	NSLBP-Low A /Control	1.00	NSLBP-Low A /Control	0.35
	Control	2.08(0.18)	2.40(0.12)	NSLBP-High A/ NSLBP-Low A	1.00	NSLBP-High A/ NSLBP-Low A	0.02*
TTS- AP (s)	NSLBP-High A	2.49(0.15)	3.38(0.13)	NSLBP-High A /Control	1.00	NSLBP-High A /Control	0.00*
	NSLBP-Low A	2.52(0.11)	3.03(0.95)	NSLBP-Low A /Control	1.00	NSLBP-Low A /Control	0.00*
	Control	2.15(1.0)	2.41(0.12)	NSLBP-High A/ NSLBP-Low A	1.00	NSLBP-High A/ NSLBP-Low A	0.00*
TTS- Total (s)	NSLBP-High A	2.14(0.15)	2.96(0.57)	NSLBP-High A /Control	1.00	NSLBP-High A /Control	0.00*
	NSLBP-Low A	2.17(0.11)	2.50(0.16)	NSLBP-Low A /Control	1.00	NSLBP-Low A /Control	0.22
	Control	2.15(1.0)	2.41(0.12)	NSLBP-High A/ NSLBP-Low A	1.00	NSLBP-High A/ NSLBP-Low A	0.02*
**Postural sway**							
COP Mean Velocity (mm/s)	NSLBP-High A	49.34(17.60)	22.26(6.06)	NSLBP-High A /Control	0.67	NSLBP-High A /Control	1.00
	NSLBP-Low A	43.59(10.74)	18.26(4.51)	NSLBP-Low A /Control	1.00	NSLBP-Low A /Control	0.12
	Control	43.12(12.04)	23.20(8.18)	NSLBP-High A/ NSLBP-Low A	0.78	NSLBP-High A/ NSLBP-Low A	0.29
COP AP (mm)	NSLBP-High A	108.41(49.78)	40.02(18.38)	NSLBP-High A /Control	0.16	NSLBP-High A /Control	0.11
	NSLBP-Low A	80.01(47.89)	35.94(17.57)	NSLBP-Low A /Control	1.00	NSLBP-Low A /Control	0.09
	Control	74.36(42.99)	56.98(27.68)	NSLBP-High A/ NSLBP-Low A	0.31	NSLBP-High A/ NSLBP-Low A	1.00
COP ML (mm)	NSLBP-High A	34.62(17.02)	19.78(9.75)	NSLBP-High A /Control	1.00	NSLBP-High A /Control	1.00
	NSLBP-Low A	30.23(15.29)	22.03(13.10)	NSLBP-Low A /Control	0.44	NSLBP-Low A /Control	1.00
	Control	38.60(14.34)	22.87(8.49)	NSLBP-High A/ NSLBP-Low A	1.00	NSLBP-High A/ NSLBP-Low A	1.00
RMS Total (mm)	NSLBP-High A	118.28(47.92)	48.68(17.82)	NSLBP-High A /Control	0.22	NSLBP-High A /Control	0.05*
	NSLBP-Low A	90.94(44.52)	46.65(17.65)	NSLBP-Low A /Control	1.00	NSLBP-Low A /Control	0.02*
	Control	89.15(38.56)	66.90(25.11)	NSLBP-High A/ NSLBP-Low A	0.28	NSLBP-High A/ NSLBP-Low A	1.00

AP: anteroposterior, COP: center of pressure, DVJ: double-leg vertical drop jump, ML: mediolateral, NSLBP-High A: nonspecific low back pain group with high pain-related anxiety, NSLBP-Low A: nonspecific low back pain group with low pain-related anxiety, RMS: root mean square, SVJ: single-leg vertical jump, TTS: time to stabilization

There was a significant difference between groups for the TTS measures during DVJ. For the total, AP, and vertical TTS, the time taken to recover stability was longer for the NSLBP group with high pain-related anxiety compared to both the NSLBP group with low pain-related anxiety and the control groups. Only the TTS in the AP direction was longer in the group of NSLBP with low pain-related anxiety compared to the control group (Table 2).

#### COP parameters

Significant differences between groups were observed only for total RMS distance during the DJV task. Post hoc analysis revealed greater total RMS distance in the control group compared to both NSLBP groups with high and low pain-related anxiety. None of the COP parameters were different between the groups during SVJ (Table 2).

## Discussion

In this study, the dynamic postural control was assessed in athletes with and without NSLBP with high and low pain-related anxiety. According to the results, during DJV, NSLBP athletes with high pain-related anxiety tended to display longer TTS in all total, AP, and ML directions compared with other groups. This means that these athletes showed poorer postural control than the other two groups. Also, during DJV, the control group revealed greater total RMS distance compared to both NSLBP groups. However, during SVJ, no significant differences were found between the groups in any of the TTS and COP parameters.

In this study, athletes with NSLBP and high pain-related anxiety showed longer TTS during DJV compared to other groups in all assessed directions. This result provides evidence for a central effect of pain-related anxiety on the sensory-motor system. During the DJV task, the participants jumped down from a height, which can create the perception of threat [48, 49]. Anxiety facilitates the early detection of threats [50], which can affect the control of posture during DJV. Psychological factors are associated with changes in landing control; so people who have more anxiety, compensate for their landing by reducing its speed [49]. This slowdown strategy is known as the protective postural strategy [51].

NSLBP athletes with high pain-related anxiety seem to need more time to land and return to a stable posture. It seems that the central nervous system of athletes with NSLBP and high pain-related anxiety strengthens the possibility of falling and leads to slowing down their landing pattern. This cautious behavior and increased duration of a movement were also observed in less dynamic tasks, such as rising to toes while standing at the edge of an elevated surface [52]. The confounding effects of psychological factors on postural control during landing and standing on elevated surfaces have been consistently reported in previous studies [49, 53, 54].

Higher TTS measures in the AP direction were observed in both NSLBP groups with high and low pain-related anxiety compared to the control. In this regard, previous studies have shown impaired neuromuscular control and proprioception deficits of the lumbopelvic region in LBP individuals which might impact the control of trunk control in the AP direction [55, 56]. The recovery from the instability during the sudden transition from dynamic to static state of landing is more prominent in the sagittal plane. This stability requires precise neuromuscular control of the trunk, which is impaired in individuals with LBP [56].

During the DJV task, the control group revealed greater total RMS distance compared to both NSLBP groups with high and low pain-related anxiety. Sufficient neuromuscular control is required to maintain balance during the transition from a dynamic to a static state. Greater postural sway observed in healthy individuals may indicate exploratory behavior as a result of perceived instability as a consequence of this transition. Increased postural sway can be used by the central nervous system as an exploratory mechanism to ensure that continuous dynamic inputs are provided by multiple sensory systems during the static positions. This increased body sway is suggested to be a perceptual-action strategy that allows us to gather essential information and essential components of postural control for tracking the body’s position relative to its stability limits [57]. However, it seems subjects in the NSLBP groups with high and low pain-related anxiety have altered the postural control strategy to protect the spine through adopting a stiffening strategy. Consistent with our findings, a reduced COP range has been reported in studies assessing the effects of unpleasant emotions on postural control [58, 59] as well as in experiments that use postural threat situations to induce anxiety [54, 59].

The present study, found a significant difference between groups in TTS and COP parameters only during DVJ, while during SVJ, there was no significant difference in any of the TTS and COP parameters. It has been postulated that balance performance in various dynamic balance tests cannot be considered interchangeable. It is consistent with the results of a previous study that divided 36 balance tests into six categories and compared them. The results showed participants with balance deficiencies in one category didn’t necessarily score poorly in other categories. As a result, they admitted that the results of cross-sectional studies depend on the type of test as well as on the sport and completion level of the athlete [60].

### Limitations

The present study has some limitations. First, only women athletes were recruited; therefore, the results can’t be generalized to male athletes. Second, although valid and reliable questionnaires were used, their self-reported nature might have caused recall bias. Finally, the study’s cross-sectional design limits the evidence level of the findings.

## Conclusion

The results of this study indicate athletes with NSLBP and higher levels of pain-related anxiety showed longer TTS measures and as a result poor postural control in comparison with NSLBP with low pain-related anxiety and the control group during the DJV task. Also, the control group revealed greater total RMS distance compared to both NSLBP groups with high and low pain-related anxiety during the DJV task. The findings suggest that pain-related anxiety could be considered as a contributing factor for the postural control strategy selected by the athletes. Therefore, it seems essential to consider the level of pain-related anxiety during planning postural control exercises for athletes with NSLBP. Also, considering the effect of gender on anxiety [61], it is recommended future studies perform the study with men to compare both genders’ data.

## Data Availability

The data presented in this study are available on reasonable request from the corresponding author.

## Abbreviations

ANOVA: Analysis of variance
AP: Anteroposterior
BMI: Body Mass Index
COP: Center of pressure
DVJ: Double-leg vertical drop jump
GRF: Ground reaction forces
LBP: Low back pain
ML: Mediolateral
NSLBP: Nonspecific low back pain
PASS-20: Pain Anxiety Symptom Scale-20
RMS: Root mean square
STAI: State-trait anxiety inventory questionnaire
SVJ: Single-leg vertical jump
TTS: Time to stabilization
VAS: Visual analogue scale
